# Deep Kalman Filter: Simultaneous Multi-Sensor Integration and Modelling; A GNSS/IMU Case Study

**DOI:** 10.3390/s18051316

**Published:** 2018-04-24

**Authors:** Siavash Hosseinyalamdary

**Affiliations:** Department of Earth Observation Science (EOS), Faculty of Geo-information Science and Earth Observation (ITC), University of Twente, Enschede 7514AE, The Netherlands; s.hosseinyalamdary@utwente.nl; Tel.: +31-(0)53-489-3921

**Keywords:** deep Kalman filter, Simultaneous Sensor Integration and Modelling (SSIM), GNSS/IMU integration, recurrent neural network (RNN), deep learning, Long-Short Term Memory (LSTM)

## Abstract

Bayes filters, such as the Kalman and particle filters, have been used in sensor fusion to integrate two sources of information and obtain the best estimate of unknowns. The efficient integration of multiple sensors requires deep knowledge of their error sources. Some sensors, such as Inertial Measurement Unit (IMU), have complicated error sources. Therefore, IMU error modelling and the efficient integration of IMU and Global Navigation Satellite System (GNSS) observations has remained a challenge. In this paper, we developed deep Kalman filter to model and remove IMU errors and, consequently, improve the accuracy of IMU positioning. To achieve this, we added a modelling step to the prediction and update steps of the Kalman filter, so that the IMU error model is learned during integration. The results showed our deep Kalman filter outperformed the conventional Kalman filter and reached a higher level of accuracy.

## 1. Problem Statement

Global Navigation Satellite Systems (GNSS) enable us to locate ourselves within a few centimeters all over the world. This system consists of a Global Positioning System (GPS), Galileo, GLobal Orbiting NAvigation Satellite System (GLONASS), and Beidu, and it is integrated into our daily lives, from car navigators to airplanes. Unfortunately, GNSS positioning requires a clear sky view; therefore, it is not available in urban canyons where GNSS signals are blocked by high-rise buildings. Other alternative navigation solutions have been applied to overcome this shortcoming of GNSS positioning and bridge its gaps in urban canyons.

Among alternative navigation solutions, inertial navigation and visual odometry are cost-effective and do not require any infrastructure. The Inertial Measurement Unit (IMU) is a composition of accelerometers and gyroscopes and it estimates the position, velocity, and orientation of a platform from measured accelerations and angular rates. 

The error characteristics of IMU sensors are complicated. They differ significantly from one technology, one manufacturer, and even one sensor, to another. The cold atom is the most accurate IMU [1], but it is very expensive and not applicable for commercial applications, such as mobile mapping. Other IMU technologies, such as mechanical IMU and Micro-Electro-Mechanical System (MEMS), suffer from common error sources, such as bias and scale factor errors and some technology-specific error sources, such as dead zone errors in Ring Laser Gyros (RLGs). 

IMU sensors have systematic, random, and computational error sources. IMU manufacturers use controlled environments, such as turntable, to estimate and remove systematic errors. The calibration procedures associated with controlled environment are costly and it cannot fully remove systematic errors. The systematic and random errors of IMU can be estimated if the relationship between these errors and the observations are known. When GNSS positioning is available, these errors are approximated using pre-existing IMU error models and this process is called IMU calibration. When IMU errors are estimated, they are removed from IMU observations and IMU positioning becomes more accurate. Unfortunately, the pre-existing models of IMU errors are not accurate and they are based on some statistical assumptions that may not hold. 

In addition, Bayes filters, such as the Kalman and particle filters, have some limitations that may introduce computational error. For instance, the conventional Kalman filter is linear and cannot handle non-linear error sources. Therefore, if the conventional Kalman filter is applied for non-linear IMU error modelling, it leads to computational error. Variants of the Kalman filter and particle filter have been developed to overcome the shortcomings of the conventional Kalman filter, but they are also limited to statistical assumptions. 

If the error characteristics of IMU sensors can be accurately modelled, the accuracy of IMU positioning can be significantly improved. Currently, scientists suggest modelling error sources of IMU separately. For instance, Reference [2] suggested modelling accelerometer and gyroscope bias as a random constant, and [3] applied the first-order Gauss–Markov stochastic process to model these biases. 

In this paper, we introduce the deep Kalman filter to simultaneously integrate GNSS and IMU sensors and model IMU errors. In contrast to previously proposed approaches, our approach does not have any pre-defined IMU error model and it is learned from observations. Therefore, we do not need to assume any stochastic or deterministic behavior of IMU errors. In contrast to previously proposed approaches, we can accurately model non-linear, time-variant, highly correlated IMU error sources.

### 1.1. Literature Review

There are a few scientific endeavors to harness IMU errors and provide accurate alternative positioning systems in the absence of GNSS positioning. As we have already discussed, accelerometer and gyro bias were modelled as random constants in [2], and they were modelled as first-order Gauss–Markov stochastic processes in [3]. Shin and El-Sheimy [4] used gravity and the earth rotation rate to calibrate IMU errors. 

Wang et al. [5] treated IMU error sources as a time series and applied the AutoRegressive and Moving Average (ARMA) method to model IMU error sources. In addition to the statistical estimators, shallow Multi-Layer Perceptron (MLP) networks have been utilized to model IMU error sources in the presence of GNSS positioning [6,7]. Adaptive Neural Fuzzy Information Systems (ANFIS) have also been applied to capture IMU uncertainties [8,9]. Toth and colleagues applied neural networks and fuzzy logic [10] to model IMU error sources. Navidi et al. [11] used hybrid Fuzzy Inference Systems (FIS) and the second-order extended Kalman filter to integrate GNSS and IMU sensors. Xing and colleagues showed that chaotic particle swarm optimization significantly reduced gyroscope random drift [12]. Applied neural networks are shallow and they do not accurately calibrate IMU since they do not consider the IMU’s correlation over time. In other words, IMU error sources should be studied as a time series and they should be modelled over time. 

It has been shown that shallow neural networks can only model simple phenomena and that complicated systems should be modelled using deep neural networks [13]. Therefore, we applied deep neural network for sensor integration. To our knowledge, we are the first to apply deep neural networks for GNSS and IMU integration and IMU error modelling. 

Mirowski and Lecun [14] introduced dynamic factor graphs and reformulated Bayes filters as recurrent neural networks. In their proposed approach, the observation and system models of the Kalman filter are learned from observations. Gu et al. [15] reformulated the Kalman filter and recurrent neural network to model face landmark localization in videos. Krishnan et al. [16] integrated the Kalman filter and variational methods for learning famous handwritten digit datasets, Modified National Institute of Standards and Technology (MNIST).

### 1.2. Kalman Filter

The unknown vector, which is estimated in the Kalman filter, is called a state vector and it is represented by $x\in {\mathbb{R}}^{n}$, where *t* indicates the state vector at time *t*. It also depends on the observation vectors, $z_{1:t}$, where $z\in {\mathbb{R}}^{m}$, and the initial state of the system $x_{0}$. The probability of the state vector at the current time is $\mathrm{Pr}(x_{t}|z_{1:t},x_{0})$. Therefore, the current state vector is estimated using Maximum Likelihood (ML) estimation, such that:

(1) $${\hat{x}}_{t}=\underset{x_{t}}{\mathrm{argmax}}\mathrm{Pr}(x_{t}|z_{1:t},x_{0})$$

The probability of the current state vector depends on the previous state vectors, such that:

(2) $${\hat{x}}_{t}=\underset{x_{t}}{\mathrm{argmax}}\frac{\mathrm{Pr}(z_{t}|x_{t})\mathrm{Pr}(x_{t}|z_{1:t-1},x_{0})}{\mathrm{Pr}\left(z_{1:t}\right)}$$

The denominator in Equation (2) is a normalization constant and it does not contribute to the maximization of likelihood in Equation (2), such that:

(3) $${\hat{x}}_{t}=\underset{x_{t}}{\mathrm{argmax}}\mathrm{Pr}(z_{t}|x_{t})\mathrm{Pr}(x_{t}|z_{1:t-1},x_{0})$$

The state vector at the current time directly depends on the previous state vectors. Therefore, the marginalization of previous state vectors is applied and leads to state vector estimation based on previous state vectors, such that:

(4) $${\hat{x}}_{t}=\underset{x_{t}}{\mathrm{argmax}}\mathrm{Pr}(z_{t}|x_{t})\int \mathrm{Pr}(x_{t}|x_{1:t-1})\mathrm{Pr}(x_{1:t-1}|z_{1:t-1},x_{0})dx_{1:t-1}$$

Based on the Markovian assumption, the state vector at the current time only depends on the state vector at previous times, such that:

(5) $${\hat{x}}_{t}=\underset{x_{t}}{\mathrm{argmax}}\mathrm{Pr}(z_{t}|x_{t})\int \mathrm{Pr}(x_{t}|x_{t-1})\mathrm{Pr}(x_{t-1}|z_{1:t-1},x_{0})dx_{t-1}$$

where $\mathrm{Pr}(z_{t}|x_{t})$ is the posterior probability estimation of the current state vector. The state vector best estimate in the previous time is ${\hat{x}}_{t-1}=\underset{x_{t-1}}{\mathrm{argmax}}\mathrm{Pr}(x_{t-1}|z_{1:t-1},x_{0})$. Therefore, Equation (5) is reformulated as:

(6) $${\hat{x}}_{t}=\underset{x_{t}}{\mathrm{argmax}}\mathrm{Pr}(z_{t}|x_{t})\int \mathrm{Pr}(x_{t}|x_{t-1}){\hat{x}}_{t-1}dx_{t-1}$$

where $\int \mathrm{Pr}(x_{t}|x_{t-1}){\hat{x}}_{t-1}dx_{t-1}$ is the prior probability estimation. This predicts the current state vector based on the system model and the posterior estimation of the state vector at previous times.

In the Kalman filter, the state vector is related to the state vector at previous times, *t* − 1, using the system model, *f*, such that:

(7) $$x_{t}=f\left(x_{t-1}\right)+\;\epsilon_{t}$$

where $\epsilon_{t}$, is the noise of the system model. The state vector relates to the observation vector, $\;z_{t}\in {\mathbb{R}}^{m}$, with the observation model, *g*, such that:

(8) $$z_{t}=g\left(x_{t}\right)+\;\omega_{t}$$

where *ω_t_*, is the noise of the observation model. The state and observation models are assumed to be linear in the Kalman filter. Therefore, these functions can be replaced by *F* and *G* matrices, respectively. The system model can be rewritten as:

(9) $$x_{t}=Fx_{t-1}+\;\epsilon_{t}$$

and similarly, the observation model can be rewritten as:

(10) $$z_{t}=Gx_{t}+\;\omega_{t}$$

The noise of the system and observation models are also assumed to have a Normal distribution in the Kalman filter, such that:

(11) $$\epsilon_{t}\sim N\left(0,Q_{t}\right)$$

(12) $$\omega_{t}\sim N\left(0,R_{t}\right)$$

where $Q_{t}$ and $R_{t}$ are the covariance matrices of the system and observation models.

The Kalman filter is composed of two-stage optimization. In the first stage, the current state vector is predicted based on the state vector in the previous time, such that:

(13) $$x_{t}^{-}=Fx_{t-1}^{+}$$

The predicted values are represented by the superscript ‘−’ and the updated values are represented by the superscript ‘+’. Error propagation is applied to estimate the covariance matrix of the current state vector based on the covariance matrix of the state vector in the previous time, such that:

(14) $$P_{t}^{-}=FP_{t-1}^{+}F+Q_{t}$$

where $P_{t}^{-}$ is the predicted covariance matrix of the state vector. 

In the update stage of the Kalman filter, the current state vector is updated by using the observation vector, such that:

(15) $$x_{t}^{+}=x_{t}^{-}+K_{t}\left(z_{t}-G_{t}x_{t}^{-}\right)$$

and the covariance matrix of the updated current state vector is calculated, such that:

(16) $$P_{t}^{+}={\left(I-K_{t}G_{t}\right)}^{T}P_{t}^{-}\left(I-K_{t}G_{t}\right)+K_{t}{}^{T}R_{t}K_{t}$$

where $K_{t}$ is the Kalman gain matrix, calculated as:

(17) $$K_{t}=P_{t}^{-}G_{t}{}^{T}{\left(G_{t}P_{t}^{-}G_{t}{}^{T}+R_{t}\right)}^{-1}$$

**Figure 1:**
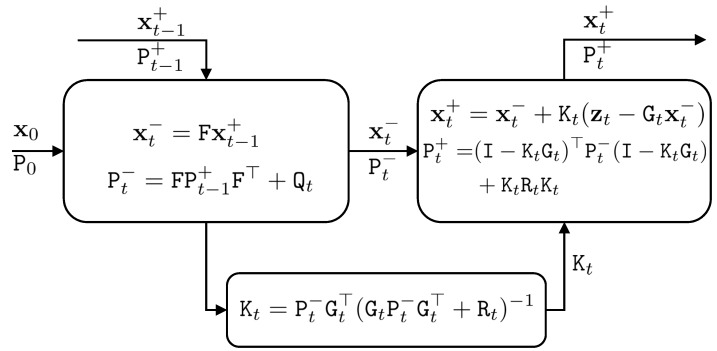
The Kalman filter procedure, which consists of prediction (**left-up box**) and update steps (**right-up box**).

For derivation of the Kalman filter equations, the reader is referred to [2]. Figure 1 shows the Kalman filter.

#### Shortcomings of the Kalman Filter

There are a number of shortcomings of the Kalman filter. Namely, *F* and *G* are linear models with Gaussian noise. Therefore, they cannot model non-linear functions or linear functions with non-Gaussian noise. In addition, these functions are time invariant, meaning they do not change over time. A number of variations in the Kalman filter, such as extended and unscented Kalman filters, can handle non-linear observation and system models. In addition, the particle filter works on observation and system models with other distributions than Gaussian noise.

Nonetheless, the observation and system models of Bayes filters should be known beforehand. In other words, scientists should find models to relate state vectors to previous state vectors and observations. As an example, we discussed that accelerometer and gyro bias can be modelled in different ways. Unfortunately, the observation and system models cannot be determined beforehand in many applications.

Another shortcoming of Bayes filters is their Markovian assumption. In Bayes filters, it is assumed that the current state vector only depends on the previous state vector and it is independent from older state vectors. Although this property significantly simplifies the system model and makes these filters very efficient, it makes the Bayes filter insensitive to system behavior with longer correlation times. In other words, complicated error models with long correlation times cannot be modelled using Bayes filters.

Here, we provide our discussion with an example of a case study on IMU error modelling. IMU models have different error sources depending on the applied technology in the accelerometer and gyroscope of the IMU. One IMU manufacturer can calibrate IMU error sources different to another manufacturer. Moreover, the error sources of MEMS sensors can significantly differ from one MEMS sensor to another, even in one MEMS family. Pre-defined IMU error models cannot handle the high variation of error sources in MEMS sensors. In addition, the high correlation between different MEMS error sources makes IMU error modelling very complicated. Often, it is not possible to discriminate error sources in MEMS sensors.

## 2. Methodology

In this paper, we add system modelling to the Kalman filter and refer to it as the deep Kalman filter. In other words, the deep Kalman filter is able to estimate the system model and it is useful in many applications, such as GNSS/IMU integration, where the system model is complicated. In order to estimate the system model of the Kalman filter, we add latent variables to the Kalman filter. Latent variables are not observed and they are invisible in the state vector, but the state vector depends on the latent variables. As an example, IMU errors depend on temperature, but this is not observed and cannot be estimated. These variables are represented by the latent vector, $h_{t}$, where the subscription *t* stands for the latent vector at the current time. The current latent vector depends on the previous latent vectors, $h_{t-1:t-T}$, where *T* is the number of previous latent vectors utilized for the current latent vector estimation.

We assume the current state vector, $x_{t}$, depends on the current latent vectors, $h_{t}$. Therefore, the current state vector indirectly depends on previous state vectors, $x_{t-1:t-T}$, and the Markovian assumption does not hold anymore. We design our modelling step in the way that the current state vector depends on the current latent vector and the current latent vector depends on previous state vectors and previous latent vectors. This is one of many different architectures of possible networks, but it significantly simplifies our network.

Let us assume there is a function ϕ that relates the current latent vector to the previous latent vectors and previous state vectors, such that:

(18) $$h_{t}=\phi \left(x_{t-1:t-T}^{+},h_{t-1:t-T}\right)$$

and the current state vector is directly related to the current latent vector by a function, λ, such that:

(19) $$x_{t}^{+-}=\lambda \left(h_{t}\right)+\mu_{t}$$

we name the posterior estimation based on our model as $x_{t}^{+-}$. In other words, $\;x_{t}^{+-}$ is the predicted posterior estimation of the state vector. We can approximate *φ* and *λ* with a combination of linear and non-linear functions. Linear functions can be represented by matrix multiplication, where coefficients of linear functions are represented by coefficient matrices, *W*. The non-linear function, σ, has no coefficient. Therefore, our network is designed, such that:

(20) $$h_{t}=\sigma \left(W_{xh}x_{t-1:t-T}^{+},W_{hh}h_{t-1:t-T}\right)$$

(21) $$x_{t}^{+-}=\sigma \left(W_{xx}h_{t}\right)+\mu_{t}$$

**Figure 2:**
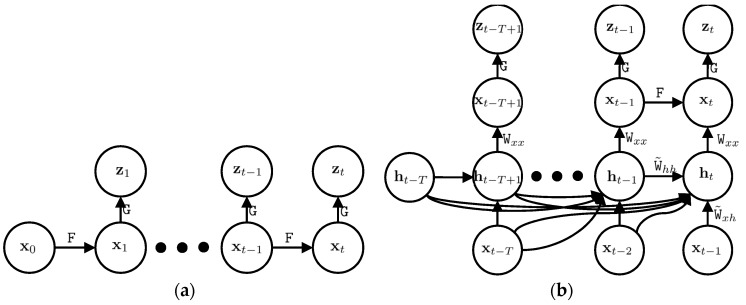
The probabilistic graphical model of the Kalman filter (**a**) and deep Kalman filter (**b**); *x*, *z*, and *h* are the state vector, observation vector, and latent vector, respectively. The matrices F and G are the system model and observation model of the Kalman filter and W is the coefficient matrix of our proposed IMU model. The two upper layers of the deep Kalman filter are similar to the Kalman filter and the added two lower layers enable the deep Kalman filter to estimate our system model.

Let us name the coefficients of the latent vector as $W_{h}$, where $W_{h}=\left[W_{xh},W_{hh}\right]$. In order to model our system, it suffices to estimate $W_{h}$ and $W_{xx}$. Figure 2 shows the probabilistic graphical model of the Kalman filter and deep Kalman filter. The upper part of the deep Kalman filter is the prediction and update steps and it is similar to the conventional Kalman filter. However, we added a modelling step to the Kalman filter, which is the lower part of the deep Kalman filter in Figure 2.

### 2.1. Expectation Maximization

When the parameters of the system model are unknown, in addition to the state vector, the system model and state vector cannot be directly estimated. In other words, the state vector depends on the latent variables of the system model and therefore, it is not estimable. In order to find the state vector and latent vector, an expectation maximization approach is utilized [17]. Expected Maximization is an iterative approach to estimate the latent vectors, as well as the state vector. In the first step, the latent vector is guessed, represented by $\;h_{t}^{\left(0\right)}$, where superscript 0 shows that this is the initial guess of the latent vector. The state vector is estimated based on the guessed latent vector, such that:

(22) $${\hat{x}}_{t}^{\left(1\right)}=\arg\underset{x_{t}}{\max}\mathrm{Pr}(x_{t}|h_{t}^{\left(0\right)},z_{1:t},x_{0})$$

In contrast to Equation (1), where the state vector is estimated based on a pre-defined system model, Equation (22) obtains the state vector based on the guessed latent vector. In the next step, the latent vector is estimated based on the calculated state vector in Equation (22), such that:

(23) $${\hat{h}}_{t}^{\left(1\right)}=\arg\underset{h_{t}}{\max}\mathrm{Pr}({\hat{x}}_{t}^{\left(1\right)}|h_{t}^{},z_{1:t},x_{0})$$

Using Equation (21), it suffices to estimate $W_{xx}$ in order to calculate the state vector, $x_{t}$. Similarly, it suffices to estimate $W_{h}$ in order to find the latent vector, $h_{t}$, and model our system. Replacing the coefficient matrices from Equations (20) and (21) into Equation (22), derives the coefficient matrices, such that:

(24) $${\hat{W}}_{xx}^{\left(1\right)}=\arg\underset{W_{xx}}{\max}\mathrm{Pr}(x_{t}|h_{t}^{\left(0\right)},z_{1:t},x_{0})$$

When the coefficient matrix ${\hat{W}}_{xx}^{\left(1\right)}$ is estimated, the state vector, $\;{\hat{x}}_{t}^{\left(1\right)}$, is calculated. The coefficient matrices of Equation (20) are estimated, such that:

(25) $${\hat{W}}_{h}^{\left(1\right)}=\arg\underset{W_{h}}{\max}\mathrm{Pr}({\hat{x}}_{t}^{\left(1\right)}|h_{t}^{},z_{1:t},x_{0})$$

The iterative estimation of latent and state vectors continues until the algorithm converges to its solution, the system model is determined and the state vector is estimated. Mirowski and LeCun [14] showed that expectation maximization can lead to recurrent neural networks. 

Expectation maximization will lead to a global maximum in convex functions. However, it is most likely it will converge to a local maximum, since modelling is not convex for complicated models. One of the challenges is finding an approach to maximize Equations (24) and (25). 

**Figure 3:**
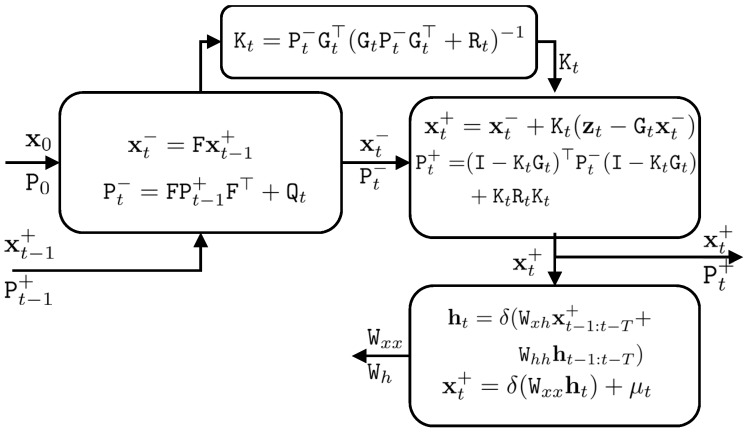
The deep Kalman filter procedure. The IMU modelling step (**right-bottom**) has been added to the Kalman filter. The modelling is accomplished in two steps: in the first step, the current latent vector is estimated based on previous latent and state vectors; and in the second step, the current state vector is estimated based on the current latent vector.

Figure 3 shows the scheme of the proposed deep Kalman filter.

### 2.2. Recurrent Neural Network

We rewrite Equation (6) based on the latent vector estimation, such that:

(26) $${\hat{x}}_{t}=\underset{x_{t}}{\mathrm{argmax}}\mathrm{Pr}(z_{t}|x_{t})\int \mathrm{Pr}(x_{t}|h_{t})\mathrm{Pr}(h|h_{t-1:t-T},x_{t-1:t-T}){\hat{x}}_{t-1:t-T}dx_{t-1:t-T}$$

In the E step of the EM algorithm, we calculate ${\hat{x}}_{t}$ based on the guessed model and the observations. In the case of GNSS and IMU integration, we can use the Kalman filter to estimate the state vector, $x_{t}^{+}$. Since GNSS observations are accurate, the estimated state vector is accurate too. In the M step of the EM algorithm, we use the guessed model to calculate the state vector and we represent the approximated state vector based on the guess model by $x_{t}^{+-}$. If our model is accurate, the approximate state vector, $x_{t}^{+-}$, should be close to the estimated state vector, $x_{t}^{+}$. Therefore, their difference is stated as an energy function, *E*, such that:

(27) $$E\left(W_{h},W_{xx}\right)=\frac{1}{2}{\left(x_{t}^{+-}-x_{t}^{+}\right)}^{2}$$

We can model our system if we minimize this energy function. By replacing $x_{t}^{+-}$ from Equation (21), Equation (26) is reformulated as:

(28) $$E\left(W_{h},W_{xx}\right)=\frac{1}{2}{\left(\sigma \left(W_{xx}h_{t}\right)-x_{t}^{+}\right)}^{2}$$

Therefore, the gradient of the coefficient matrix, $W_{xx}$, is calculated as:

(29) $$\frac{\partial E\left(W_{h},W_{xx}\right)}{\partial W_{xx}}=\frac{\partial \sigma \left(W_{xx}h_{t}\right)}{\partial W_{xx}}{\left(\sigma \left(W_{xx}h_{t}\right)-x_{t}^{+}\right)}^{}$$

The gradient of the latent vector coefficient matrix, $W_{h}$, is also estimated as:

(30) $$\frac{\partial E\left(W_{h},W_{xx}\right)}{\partial W_{h}}=\frac{\partial \sigma \left(W_{xh}x_{t-1:t-T}^{+}+W_{hh}h_{t-1:t-T}\right)}{\partial W_{h}}\frac{\partial \sigma \left(W_{xx}h_{t}\right)}{\partial W_{xx}}{\left(\sigma \left(W_{xx}h_{t}\right)-x_{t}^{+}\right)}^{}$$

In order to minimize the energy function, we use an iterative gradient descent. Therefore, the coefficient matrices of the modelled system, $\;W_{xx}$ and $W_{h}$, are estimated, such that:

(31) $$W_{xx}^{\left(m+1\right)}=-\frac{\partial E\left(W_{h},W_{xx}\right)}{\partial W_{xx}}\mu +W_{xx}^{\left(m\right)}$$

(32) $$W_{h}^{\left(m+1\right)}=-\frac{\partial E\left(W_{h},W_{xx}\right)}{\partial W_{h}}\mu +W_{h}^{\left(m\right)}$$

where μ is the learning rate. When the coefficient matrices are determined and system model is learned, $\;x_{t}^{+-}$ can be estimated based on the previous state vectors, $x_{t-1:t-T}^{+}$, in Equations (20) and (21).

### 2.3. Long Short-Term Memory

Recurrent neural networks have a drawback, known as the exploding-vanishing gradient problem [18]. When *T* is large and we model the system for a long time span, the gradients are multiplied in several layers. If the gradients are large, their multiplication becomes humongous and their gradient explodes. On the contrary, if the gradients are small, their multiplication becomes insignificant, known as a vanishing gradient. In order to prevent such an effect in recurrent neural networks, the Long Short-Term Memory (LSTM) method involves the introduction of gated memories [19]. In LSTM, the network consists of cells and each cell can memorize the previous state vectors using an input gate, remember them using an output gate, and forget them using a forget gate. Let us represent the input gates as $i_{t}$, output gates as $o_{t}$, and forget gates as $f_{t}$. They are represented by a combination of linear and non-linear functions, such that:

(33) $$f_{t}=\sigma \left(W_{f}x_{t}+U_{f}h_{t-1}\right)$$

(34) $$i_{t}=\sigma \left(W_{i}x_{t}+U_{i}h_{t-1}\right)$$

(35) $$o_{t}=\sigma \left(W_{o}x_{t}+U_{o}h_{t-1}\right)$$

where σ is the non-linear function and the linear functions are represented by the coefficient matrices, $W_{f}$, $W_{i}$, and $W_{o}$. The cell state, $c_{t}$, and latent layer, $h_{t}$ , are estimated as:

(36) $$c_{t}=f_{t}\circ c_{t-1}+i_{t}\circ \sigma \left(W_{c}x_{t}+U_{c}h_{t-1}\right)$$

(37) $$h_{t}=o_{t}\sigma \left(c_{t}\right)$$

where ∘ is a Hadamard product. For long term correlation, the input gate can keep information from previous state vectors and the gradients of previous state vectors are accessible. Therefore, the gradients do not explode or vanish in the back-propagation process. The forget gate controls the complexity of the model and it removes the uncorrelated previous state vectors.

## 3. Implementation

In this section, we explain the details of our model implementation. We first explain the implementation of our Kalman filter and then we explain the implementation of our proposed deep Kalman filter.

It has been shown that the Kalman filter works better on IMU errors than IMU output [2,7] Therefore, we utilized the IMU mechanization to estimate position, velocity, and orientation. However, IMU mechanization is not perfect and errors related to position, velocity, and orientation remain in the system. We defined the state vector of the Kalman filter in terms of positioning error, velocity error, orientation error, and the bias of accelerometers and gyroscopes. The system model utilized in the Kalman filter is similar to Reference [3] and described in the appendix. The GNSS observations are used as the observation vector and applied to the observation model to update the state vector.

We implemented an extended Kalman filter and applied the system and observation models to predict and update the state vector. When GNSS observations are available, the extended Kalman filter predicts and updates the state vector. If GNSS observations are not available, the extended Kalman filter only predicts the state vector. 

For the implementation of our deep Kalman filter, we calculated the posterior estimation of the state vectors, $x_{t}^{+}$, when GNSS observations were available. Since we used the Real-Time Kinematic (RTK) technique for GNSS positioning, our posterior estimation was anticipated to be very accurate and it could be used as a ground truth for our IMU error modelling. We used our proposed deep Kalman filter to predict the state vector and the predicted state vector using our model was $x_{t}^{+-}$. We tried to predict $x_{t}^{+-}$ as close as possible to $x_{t}^{+}$ and, therefore, we got the best estimate of the modelled IMU errors. In other words, we found a model to calculate $x_{t}^{+-}$ as an approximation of $x_{t}^{+}$ in the presence of GNSS positioning and we replaced prior estimation of the Kalman filter, $x_{t}^{-}$, to $x_{t}^{+-}$ in the absence of GNSS positioning. If the system model is accurately estimated, the predicted state vector used has higher accuracy if the proposed deep Kalman filter is applied.

**Figure 4:**
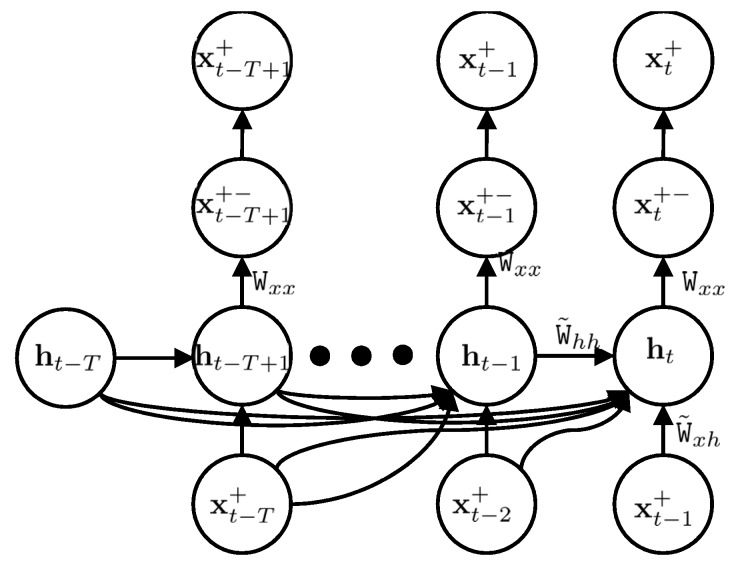
IMU error modelling reformulated as a time series prediction. The posterior estimation of the state vector is utilized in the target node (**top layer**), the output of our model is considered as the output node (**second layer**), the previous state vectors are in the input layer (**bottom layer**) and the latent vectors are in the hidden layer (**third layer**).

In order to implement the proposed approach, we applied a predictive network, where the state vector was predicted from previous state vectors and latent vectors. This predictive network is shown in Figure 4. In the predictive network, we predicted a sequence of state vectors from *t* − *T +* 1 to *t*, $x_{t-T+1}^{+},\ldots ,x_{t}^{+}\;$, based on a sequence of state vectors *t* − *T* to *t* − 1, $x_{t-T}^{+},\ldots ,x_{t-1}^{+}$. In other words, we learned the model, in addition to the state vector prediction, in every step. The sequence $x_{t-T+1}^{+},\ldots ,x_{t}^{+}\;$ was used as a target layer in our network and the sequence $x_{t-T}^{+},\ldots ,x_{t-1}^{+}\;$ was applied as an input layer. The prediction of our model was a sequence of predicted state vectors, $x_{t-T}^{+-},\ldots ,x_{t-1}^{+-}\;$. The hidden layer had a sequence of $h_{t-T},\ldots ,\;h_{t}$. We calculated the coefficient matrices $W_{xx}$ and $W_{h}$ in order to model the IMU errors.

In order to implement an efficient predictive recurrent neural network, we designed a network with only one latent layer while every latent vector, $h_{t}$, contained 3000 variables, known as nodes, in neural network terminology. We were benefited by mini-batch learning, where sequences are added together and processed once. The batch size of our networks was 800. This speeds up the learning process and improves learning convergence. Another important factor of training in recurrent neural networks is the learning rate. We used 0.05 as the initial learning rate, with 5 × 10^−3^ learning rate decay. We tried several sequence lengths and studied the impact of sequence length on accurate error modeling. 

When GNSS observations are not available, the posterior estimation of the state vector cannot be calculated. Therefore, $x_{t}^{+}$ is not estimable and we can only predict the state vector. There are two ways to predict the current state vector: using the system model of the Kalman filter or applying the modelled system model of IMU in the deep Kalman filter. In the first approach, the state vector is predicted based on a pre-defined system model, as in Equation (13). In the second approach, the predicted state vector is estimated based on our learned IMU model, as in Equations (20) and (21). We anticipated that the modelled system in the deep Kalman filter would perform better than the system model of the Kalman filter. In other words, the modelled IMU errors were anticipated to be more accurate than the predicted IMU errors of the Kalman filter.

## 4. Experiment

**Figure 5:**
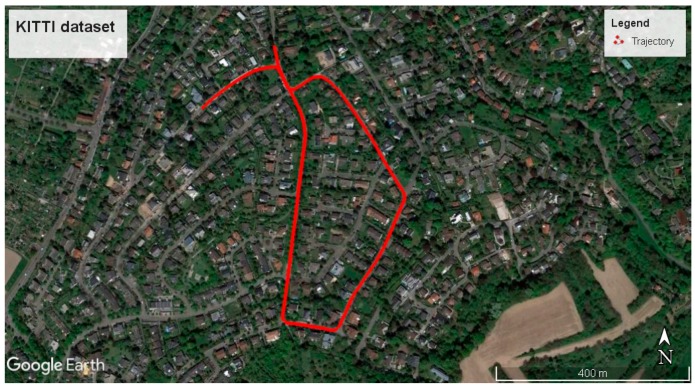
The trajectory of the KITTI dataset, #34. It is the longest KITTI dataset where the vehicle travels more than 1.7 km.

In order to evaluate our proposed approach, the KITTI benchmark [20] was utilized. This benchmark contains multi-sensor datasets and labeled information. Among various sensors, GPS and IMU information was used in this paper. We used the longest KITTI dataset, dataset No. 34, collected on 3 October 2011. This dataset contains 7:46 min of data collection, mostly driven in residential areas with a clear sky view. The trajectory of the platform exceeds 1.7 km, as shown in Figure 5. The OXTS RT 3003 navigation system was applied to collect the GPS and IMU information. The GPS and IMU information had already been synchronized and collected in 10 Hz. Manufacturers had performed calibration procedures and the calibration parameters, such as level-arm, were internally applied to transfer the GPS information into the IMU local frame. Dual-frequency RTK was used to estimate the accurate position of the IMU, with accuracy better than 10 cm.

We used the GPS observations as the ground truth to train our network and model IMU errors. When the weights of our network were learned and IMU errors were modelled, we estimated the IMU errors in two ways and compared them. First, we did not use GPS observations and used different approaches to predict IMU errors and correct IMU positioning. Second, we used GPS observations and calculated IMU errors. The calculated IMU errors using GPS observations were utilized to correct IMU positioning and we used it as baseline to evaluate corrected IMU positioning without using GPS observations. 

## 5. Results

In order to evaluate the results, we divided trajectory into two parts: one for training and calibration and one for testing and evaluation. In the training phase, the parameters of the system model were estimated in the extended Kalman filter, as described in the appendix. In the deep extended Kalman filter, IMU errors were modelled in addition to the prediction and update stages. In other words, we trained our network to predict the posterior estimation of IMU errors using a recurrent neural network (RNN) and consequently, model IMU errors. When the network was trained, we anticipated that we would be able to predict the posterior estimation of IMU errors and correct IMU positioning.

In the testing part, we estimated IMU errors using GNSS observations and corrected IMU positioning. We utilized the posterior estimation of the state vector as the ground truth. The extended Kalman and deep extended Kalman filters were applied to predict IMU errors without using GNSS observations and to correct IMU positioning. Corrected IMU positioning using these two approaches was compared with the ground truth and the IMU positioning error was calculated. The Root Mean Square Error (RMSE) of the IMU positioning was applied to evaluate the results of the extended Kalman filter and deep extended Kalman filter. 

**Figure 6:**
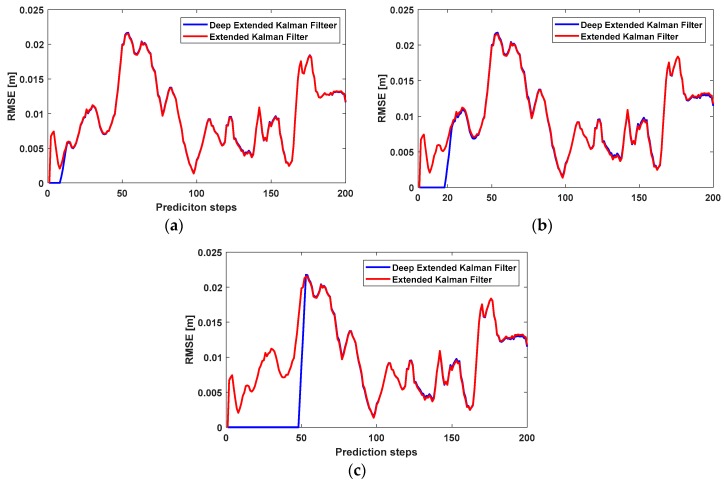
The RMSE of the deep extended Kalman filter and extended Kalman filter [21]. We used different sequence lengths of a simple recurrent neural network (RNN) for the IMU modelling of the deep extended Kalman filter. (**a**) Simple RNN with a sequence length of 10; (**b**) simple RNN with a sequence length of 20; (**c**) simple RNN with a sequence length of 50.

In the first experiment, we used a simple RNN to predict IMU errors and removed them from the IMU positioning. In this experiment, we studied the performance of the extended Kalman filter and deep extended Kalman filter using different sequence lengths. In other words, the sequences of the IMU errors, utilized to predict IMU errors in the RNN, had different lengths. We plotted the RNN for the sequence lengths of 10, 20, and 50, as shown in Figure 6. 

Figure 6 shows that the performance of RNN depends on the sequence length of previous state vectors. If the sequence length is large, the network uses more IMU errors in the previous time. In other words, the network applies more heuristics to predict IMU errors and remove them from IMU positioning. Therefore, the RMSE of the deep extended Kalman filter was less than the RMSE of the extended Kalman filter, at earlier times. 

Despite the effectiveness of RNN in the prediction of IMU errors over short periods, it cannot predict IMU errors over a long period of time. The RMSE of the deep extended Kalman filter was lower than the RMSE of the extended Kalman filter at earlier times, but the deep extended Kalman filter lost its effectiveness and the two approaches had the same RMSE over a longer period of time. This effect was due to the vanishing-exploding gradient problem of RNN. This well-known problem of RNN has been overcome by the development of Long Short-Term Memory (LSTM). LSTM uses memory gates to remember previous IMU errors and prevents repetitive gradient multiplications.

**Figure 7:**
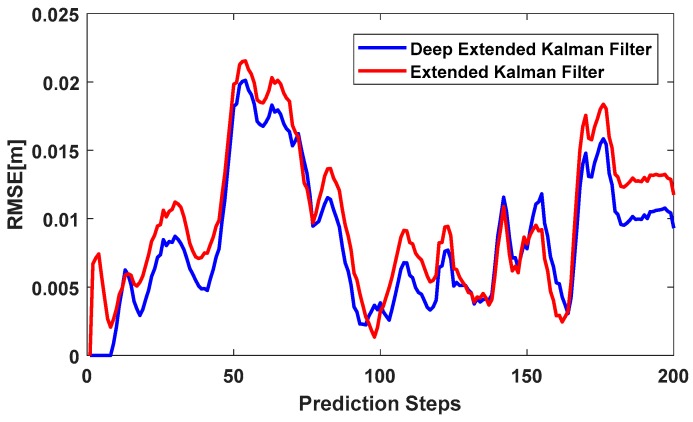
RMSE of the deep extended Kalman filter and extended Kalman filter. The deep extended Kalman filter IMU modelling was based on LSTM with a sequence length of 10.

Figure 7 shows IMU positioning using the LSTM network, with a sequence length of 10. In other words, instead of using RNN to predict IMU errors, we used LSTM to predict IMU errors and remove them from IMU positioning. The sequence length of this network was similar to Figure 6a. However, the effectiveness of this network did not disappear over a longer time period. As shown in Figure 7, the deep extended Kalman filter outperformed the extended Kalman filter the majority of the time. There were instances where the extended Kalman filter had better accuracy than the deep extended Kalman filter, but these were limited to a few short instances. The total RMSE of the deep extended Kalman filter was 0.0100 m and the total RMSE of the extended Kalman filter was 0.0114 m for 20 s. In summary, the deep extended Kalman filter had better accuracy compared to the extended Kalman filter. 

Zhang et al. [22] showed that the computational complexity of simple RNN depends on the depth of the network and the sequence length of the heuristics. Our RNN model had one layer and, roughly speaking, the complexity of the network was linearly proportional to the sequence length of the RNN for the one-layer network. In other words, training the network with a sequence length of 50 was five times slower than training the same network with a sequence length of 10. We experienced a little overhead for longer networks. LSTM is slow, therefore there are a few variants of LSTM, aimed to speed up its training step. We utilized the fast LSTM algorithm provided by Torch library in this paper. 

## 6. Conclusions

In this paper, we introduced the deep Kalman filter to learn the system model of the Kalman filter. In addition to the prediction and update steps of the Kalman filter, we added a modelling step to the deep Kalman filter. We applied the deep Kalman filter to model IMU errors and correct IMU positioning. In the deep Kalman filter, the model of IMU errors was learned using the Recurrent Neural Network (RNN) and Long Short-Term Memory (LSTM) methods when GNSS observations were available. IMU errors could be predicted using the learned model in the absence of GNSS observations.

We compared the Kalman and deep Kalman filters using the KITTI dataset. The results showed that our approach outperformed the conventional Kalman filter and a higher level of accuracy was achieved using the deep Kalman filter. In addition, the deep Kalman filter using the RNN method predicted IMU errors over short time periods, but its effectiveness disappeared over longer time periods due to the vanishing-exploding gradient problem. Employing the deep Kalman filter based on the Long Short-Term Memory (LSTM) method predicted IMU errors over longer periods of time and outperformed the Kalman filter.

## Appendix A. System and Observation Models of Kalman Filter

The applied IMU model in the Kalman filter, described in this paper is as follows. The state vector is:

(A1) $$x_{t}={\left[\delta p^{T}\;\delta v^{T}\;\delta \theta^{T}\;b_{acc}{}^{T}\;b_{gyro}{}^{T}\right]}_{t}^{T}$$

where  δp is the IMU positioning error,  δv is the IMU velocity error,  δθ is the IMU orientation error, and $b_{acc}$ and $b_{gyro}$ are accelerometer and gyroscope bias. The system model is similar to [3], as follows:

(A2) $$F=\left[\begin{array}{ccc} \begin{array}{c} 0\\ 0\\ \begin{array}{c} 0\\ 0\\ 0 \end{array} \end{array} & \begin{array}{c} D\\ 0\\ \begin{array}{c} K\\ 0\\ 0 \end{array} \end{array} & \begin{array}{ccc} \begin{array}{c} 0\\ F\\ \begin{array}{c} 0\\ 0\\ 0 \end{array} \end{array} & \begin{array}{c} 0\\ 0\\ \begin{array}{c} R\\ -\beta_{acc}\\ 0 \end{array} \end{array} & \begin{array}{c} 0\\ R\\ \begin{array}{c} 0\\ 0\\ -\beta_{gyro} \end{array} \end{array} \end{array} \end{array}\right]$$

where  0 is a  3×3 zero matrix. $\;D=\left[\begin{array}{ccc} 0 & \frac{1}{R_{M}+h} & 0\\ \frac{1}{(R_{N}+h)cos\varphi } & 0 & 0\\ 0 & 0 & 1 \end{array}\right]$ is the transformation between a geographic coordinate system to a linear coordinate system, where *h* is the ellipsoidal height and $R_{M}$ and $R_{N}$ are the curvature radiuses of the meridian and prime vertical. $\;F=\left[\begin{array}{ccc} 0 & f_{u} & -f_{n}\\ -f_{u} & 0 & f_{e}\\ f_{n} & -f_{e} & 0 \end{array}\right]$ is a skew-symmetric matrix, where its components are acceleration in east, north, and up. The rotation matrix is shown by R; $\beta_{acc}$ and $\beta_{gyro}$ are the coefficients of the first-order Gauss–Markov model for the accelerometer and gyroscope bias; and $D=\left[\begin{array}{ccc} 0 & \frac{1}{R_{M}+h} & 0\\ \frac{1}{R_{N}+h} & 0 & 0\\ \frac{\tan\varphi }{R_{N}+h} & 0 & 0 \end{array}\right]$, where φ is the latitude of the IMU. When GNSS positioning is available, the observation vector is as follows:

(A3) $$\delta p_{obs}=p_{GNSS}-p_{IMU}$$

where $p_{GNSS}$ is the GNSS positioning and $p_{IMU}$ is the IMU positioning, estimated by IMU mechanization. The observation model is calculated, such that:

(A4) $$G=\left[\begin{array}{ccc} I & 0 & \begin{array}{ccc} 0 & 0 & 0 \end{array} \end{array}\right]$$

where  I is 3 × 3 identity and 0 is 3 × 3 zero matrices.
